# The Art Amateur

**Published:** 1894-07

**Authors:** 


THE ART AMATEUR. Devoted to art in the household. Montague Marks, Publisher, 25 Union Square, New York. Price, $4.00 a year; 35 cents a number.

Volume XXX, No. 6, for May, has been received. It contains engraved copies of new paintings, notes on art exhibitions, news of galleries and studios, patterns of china decorations, and also beautiful pictures of artistic household decoration. It has pages devoted to the new publications on art. It has also two beautiful colored plates, and certainly is well worth the moderate price asked for it. Our subscribers will do well in sending thirty-five cents to the publisher, and, after they have examined the work, will find it ornamental and most useful in a home. Such works cultivate the inmates of a house and develop tastes that prove useful and productive in later years. The June number has also been received and we can hardly say enough, but we again repeat, get a number and you will want all of them as they come out. We are waiting anxiously for the July number.

